# Analysis of the hypovirulent *Klebsiella pneumoniae* with the *NDM-5* gene on IncN plasmids

**DOI:** 10.1128/spectrum.03443-23

**Published:** 2023-11-29

**Authors:** Jianhua Fang, Guoyu Wang, Xiuhua Kang, Zhenhui Pan, Yanfang Mei, Huade Chen, Yang Liu, Tianxin Xiang

**Affiliations:** 1 Department of Infectious disease, The First Affiliated Hospital of Nanchang University, Nanchang, China; 2 Department of Infectious disease, Nanchang University, Nanchang, China; 3 Department of Hospital Infection Control, The First Affiliated Hospital of Nanchang University, Nanchang, China; 4 Department of Pediatrics, Nanchang University, Nanchang, China; 5 Department of Pediatrics, The First Affiliated Hospital of Nanchang University, Nanchang, Jiangxi, China; 6 Department of Clinical Laboratory, The First Affiliated Hospital of Nanchang University, Nanchang, China; 7 Jiangxi Hospital of China-Japan Friendship Hospital, Nanchang, China; 8 Jiangxi Medical Center for Critical Public Health Events, The First Affiliated Hospital of Nanchang University, Nanchang, Jiangxi, China; University of Guelph College of Biological Science, Guelph, Ontario, Canada

**Keywords:** *NDM-5*, IncN, fitness, characteristics, *Klebsiella pneumoniae*

## Abstract

Our study aims to analyze the hypovirulent *Klebsiella pneumoniae* with the *NDM-5* gene on IncN plasmids. This strain was isolated from a patient with a liver abscess, and the ST type was ST147. The characterization, antimicrobial drug resistance, and virulence of the strain with the *NDM-5* gene on IncN plasmids were analyzed by whole-genome sequencing. Virulence testing was conducted using the Galleria mellonella infection model, serum killing, and biofilm formation assay. Growth measurement analysis, an *in vitro* competition experiment, and a plasmid stability assay were calculated using fitness costs. A conjugation assay was used to test *NDM-5* transfer between bacterial strains. For statistical analysis and image visualization, GraphPad Prism 8.0 was employed. The result revealed that the hypovirulent strain with the *NDM-5* gene was located on the IncN plasmid (92.284 kb), which is rare in *Klebsiella pneumoniae*. Whole-genome sequencing revealed the presence of 25 and 22 resistance and virulence genes, respectively. The strain KP4089 was resistant to carbapenems, β-lactam, ceftazidime-avibactam, and macrolides but remained susceptible to aminoglycosides, mucilage, and tigecycline in antimicrobial susceptibility tests. Furthermore, the strain KP4089 exhibited significantly lower serum killing and biofilm formation rates, demonstrating that the strain with the *NDM-5* gene had low virulence. The strain with the *NDM-5* gene on the IncN plasmid was genetically stable and had no fitness cost associated with identifying IncN plasmids. Future research should focus on clinical surveillance of hypovirulent multidrug-resistant *Klebsiella pneumoniae* to identify the potential strain and mechanistic studies to prevent its spread.

**IMPORTANCE:**

It is crucial to strengthen the ongoing clinical surveillance of non-highly virulent, multi-resistant *Klebsiella pneumoniae*.

## INTRODUCTION

Globally, carbapenem-resistant microorganisms are emerging as a health concern. The production of the enzyme carbapenemase by antibiotic-resistant bacteria is the mechanism of carbapenem resistance. A study by Ramsamy et al. in 2022 found that carbapenemase-encoding genes frequently share mobile genetic elements (MGEs) with a high prevalence (1). Several examples of MGEs encompass plasmids, insertion sequences, integrons, and transposons. Plasmids exhibit the capacity to penetrate cellular structures, undergo replication, reproduce, and be inherited by subsequent generations. Nonetheless, the long-term persistence of plasmids relies on the development of fitness disadvantages linked to the carrying of plasmids (2, 3).

A previous study (4) has shown that carbapenem-resistant *Enterobacteriaceae* are causing the number of carbapenem-resistant *Klebsiella pneumoniae* (CRKP) cases to rise in China. Carbapenem-resistant *Klebsiella pneumoniae* possesses *KPC*, *NDM*, and *OXA* (5). Schauer et al. (6) identified that oxacillinase (*OXA-48* and *OXA-181*) variants are the most common carbapenemases in clinically relevant *Enterobacteriaceae* strains. The ColE plasmid-located *OXA-181* gene was initially discovered in India in 2007 (7).

Currently, clinical interest has been drawn to the New Delhi metallo-β-lactamase-5 due to its high treatment resistance and rapid transmission. Mei et al. (8) state that base replacements alter the sequence of the amino acids in *NDM-5* and other antibiotics, conferring greater carbapenem resistance than other metallo-beta-lactamases. Ali et al. (9) demonstrated that *NDM-5* has a higher hydrolytic activity than *NDM-1*, *NDM-4*, *NDM-6*, and *NDM-7*. The IncX3 plasmid (10, 11) has been shown to diversify the dissemination of the *blaNDM-5* gene to non-clonal *E. coli* hosts (ST12, ST167, ST354, ST361, ST410, ST617, ST746, ST6335, and ST6395) in China. However, it is inadequately understood that a strain with the *NDM-5* gene is present in IncN plasmids. Consequently, we analyzed the strain with the *NDM-5* gene on IncN plasmids, shedding light on its characteristics and fitness cost.

## MATERIALS AND METHODS

### Isolation and antimicrobial susceptibility testing

*Klebsiella pneumoniae* (KP) was isolated from a patient with a liver abscess and then identified using a VITEK MS (Mérieux, France) automated rapid microbial mass spectrometry detection system at a tertiary hospital in Jiangxi Province.

KP4089 and its transconjugant were tested for antimicrobial susceptibility using the K–B diffusion method (Table 1). The results were interpreted according to the European Committee on Antimicrobial Susceptibility Testing guidelines. CR-hvKP30457 and *Escherichia coli* ATCC25922 were used as quality control strains.

**TABLE 1:** Antibiotic resistance profile

Agent	KP4089		4089e
	MIC	Interpretation	Interpretation
Ampicillin			
Piperacillin/tazobactam	≥128.0	R	R
Ceftazidime	≥64.0	R	R
Cefepime	≥32.0	R	R
Aztreonam	≥64.0	R	R
Imipenem	≥16.0	R	R
Meropenem	≥16.0	R	R
Amikacin	8	S	S
Ciprofloxacin	≥4.0	R	R
Levofloxacin	≥8.0	R	R
Colistin	1	S	S
Tigecycline	2	S	S

### Plasmid characterization

#### Plasmid analysis and conjugation assay

The plasmids of KP4089 were analyzed by S1-PFGE and southern blotting. In addition, a plasmid conjugation assay was performed between imipenem-resistant *Klebsiella pneumoniae* and rifampicin-resistant *Escherichia coli* EC600 to verify the transfer of plasmids containing resistance genes. Transconjugants (600 µg/mL rifampicin and 200 µg/mL imipenem) grown on the screening plate were transferred to a new screening plate, and successful plasmid transfer was verified by PCR and VITEK MS mass spectrometry.

#### Integrator analysis

The detection and identification of class 1, 2, and 3 integrons in the isolates were studied by amplifying the integrase genes intI1, intI2, and intI3 using the specific primers mentioned in Table S1.

#### Whole genome sequencing

The construction of a 10K SMRT Bell library was followed by the ligation, purification, and screening of DNA fragments using DNA adhesion enzymes, pure magnetic beads, and BluePipin fragments, respectively. Qualified DNA was divided into appropriate fragments using Covaris g-tubes. Additionally, AMPure PB magnetic beads were used to screen and purify the SMRT Bell library concentration. The size of the insert fragments was then quantified by measuring the constructed libraries with an Agilent 2100 bioanalyzer. HiSeq data were then used for sequencing, assembly, and further proofreading on the PacBio platform.

#### Comparative genomic analysis

Twenty-two strains of *Klebsiella pneumoniae* genomic sequences were analyzed by BLAST (https://blast.ncbi.nlm.nih.gov/). Whole-genome sequences for 21 strains were obtained from the NCBI website (https://www.ncbi.nlm.nih.gov/genome/). Twenty-one *Klebsiella pneumoniae* strains were included from different countries (Table 2). MEGE software was used to construct a maximum likelihood phylogenetic tree (Fig. 5).

**TABLE 2:** Comparative genomic analysis of the IncN plasmid of KP4089 with other IncN plasmids

GenBank number	Country	Max score	Total score	Query cover	*E* value	Percent identity	Accession length
CP018673.1	Britain	20,550	64,757	86%	0	98.45	44,015
CP018349.1	Britain	20,550	63,806	86%	0	98.45	43,482
CP055453.1	Britain	20,329	52,847	77%	0	96.13	41,042
CP024432.1	Sweden	15,535	59,871	84%	0	99.04	45,291
CP040996.1	America	14,462	49,076	73%	0	95.12	42,105
CP069206.1	India	13,313	45,046	69%	0	96.78	40,155
CP030069.1	Sweden	13,248	65,035	88%	0	98.05	42,064
CP084495.1	China	13,036	51,155	74%	0	96.86	43,518
CP068607.1	America	12,249	55,214	76%	0	95.3	45,912
AP021975.1	Japan	11,535	51,437	75%	0	93.66	42,597
LR890180.1	Australia	11,394	47,200	72%	0	93.11	42,522
CP064372.1	America	8,637	44,461	67%	0	93.22	45,158
AP022082.1	Japan	8,392	9,977	17%	0	93.36	41,162
CP115839.1	America	4,776	7,432	14%	0	90.1	42,434
CP094481.1	China	4,632	7,759	15%	0	89.44	43,173
CP021954.1	America	4,571	7,699	15%	0	89.12	42,816
CP035199.1	France	4,534	7,058	14%	0	88.73	43,433
CP110567.1	America	4,523	7,548	15%	0	88.36	44,274
CP083785.1	America	4,523	7,548	15%	0	88.36	44,303
CP032206.1	America	4,519	6,464	12%	0	88.82	42,202
CP110760.1	America	4,516	7,187	14%	0	88.98	43,158

### Virulence analysis

#### Serum resistance test

KP4089, the transformant, ATCC25922, and CR-hvKP NUHL 30457 were inoculated into Luria Bertani (LB) broth, shaken overnight at 37°C, and adjusted to 10^6^ CFU/mL the following day. Serum from healthy subjects and inactivated serum were added to the diluted bacterial solution and incubated, followed by applying the bacterial solution to the plates overnight. The bacterial survival rate was calculated by counting the number of colonies on both plates.

#### Biofilm assay

The biofilm formation assay was modified with reference to the method reported in the literature (12). The bacterial suspension was adjusted to 0.5 McFarland turbidity. This was followed by staining with crystalline violet, ethanol lysis, and transferring the ethanol lysate to a 96-well plate. The optical density (OD) values were recorded for each well, and the negative control values (Nc) were determined by the mean ± three times the standard deviation ( $\bar{\text{x}}$ ± 3SD). The results were evaluated as follows: strongly positive (4 × Nc < OD); positive (2 × NC < OD ≤ 4 × Nc); weakly positive (Nc < OD ≤ 2 × Nc); and negative (OD ≤ Nc).

#### Galleria mellonella infection model

The Galleria mellonella infection model was used to assess strain virulence levels (8). A total of 10 larvae weighing around 250 mg (purchased from Tianjin Huiyude Biotechnology Company, Tianjin, China) were used to test the virulence of each strain. Each was injected with 10 µl at a concentration of 1 × 10^6^ CFU/mL injection, and the survival rate of the greater wax borer was recorded every 12 h for 3 days. All experiments were performed three times. Hypervirulent *Klebsiella pneumoniae* (HvKP) strains CRKP30457 and ATCC700603 were used as controls for hyper- and hypovirulence strains, respectively.

#### String test

Bacteria were inoculated onto sheep blood agar plates for overnight incubation and a string test was performed by stretching the colonies on the plates using an inoculation loop. The string test was positive if mucus filaments > 5 mm could be pulled out, which is highly suggestive of hvKP.

### The fitness cost of the plasmid

#### Growth measurement analysis

The CR-hvKP NUHL 30457, KP4089, the transformant, and EC600 were transferred to the Bioscreen C-Pro fully automated growth curve analyzer for continuous measurement of strain OD, as previously described (13).

#### *In vitro* competition assay

We adopted the method previously described by Chen et al. (14). The transformant and EC600 were inoculated into the LB broth and incubated for 24 h. The suspension was diluted, and 10 µL of the suspension was applied to a blank LB plate and incubated overnight. Every 24 h, 10 µL of the previous day’s mixture was transferred to LB broth and incubated overnight for 4 days. Before each passaging of the strains, 10 μl of the diluted bacterial solution was taken and spread evenly on a blank LB plate; at the same time, the colonies on the blank plate were transferred to the plate containing imipenem (200 μg/ml), and the number of growing and non-growing colonies were counted separately.

#### Plasmid stability assay

The transconjugants were inoculated into LB broth and incubated for 21 days. Ten microliters of the diluted solution was evenly applied to a blank LB plate and incubated at 37°C. At the same time, the blank plate colonies were transferred to the monoclonal antibody plate containing imipenem and incubated at 37°C. The number of grown and ungrown colonies was counted separately; plasmid stability = number of grown colonies/total number of colonies. The transformants were passaged under the screening plate of imipenem for 2 weeks.

### Statistical analysis

GraphPad Prism 8.0 (windows) was used for statistical analysis and image visualization. *P* < 0.05 was considered to indicate a statistically significant difference.

## RESULTS

### Drug susceptibility testing

KP4089 exhibited resistance to carbapenems, β-lactam antibiotics, ceftazidime-avibactam, macrolides, and aminoglutethimide; however, it remained susceptible to aminoglycosides, mucilage, and tigecycline. In contrast, the transformant was resistant to many types of antibiotics, such as carbapenems, β-lactams, cephalosporins, macrolides, and aminoglutethimide. Nevertheless, the bacterium exhibited sensitivity to mucilage and tigecycline, mirroring the pre-transformant drug sensitivity. This finding provided additional evidence that the transfer of the resistant plasmid was likely to result in the development of multi-drug resistance, thus enhancing the bacteria’s resistance to treatment, as depicted in Table 1.

### Transferability of the *blaNDM-5* gene

The conjugation assay result showed that the *blaNDM-5* drug resistance gene and its resistance phenotype to imipenem were transferred to *E. coli* EC600, suggesting the *blaNDM-5* resistance gene had a mobile genetic element. However, resistance genes such as *OXA-181* and *TEM-1* in the strain were non-transferable, suggesting that the transfer of resistance genes was influenced by multiple factors and was not absolute. This finding indirectly suggests that the presence of many plasmids in bacteria could be a potential source of transmission of hypervirulent resistant genes to other bacteria, which poses a risk to antibiotics commonly used to treat human infections.

### Virulence assessment

**Fig 1:**
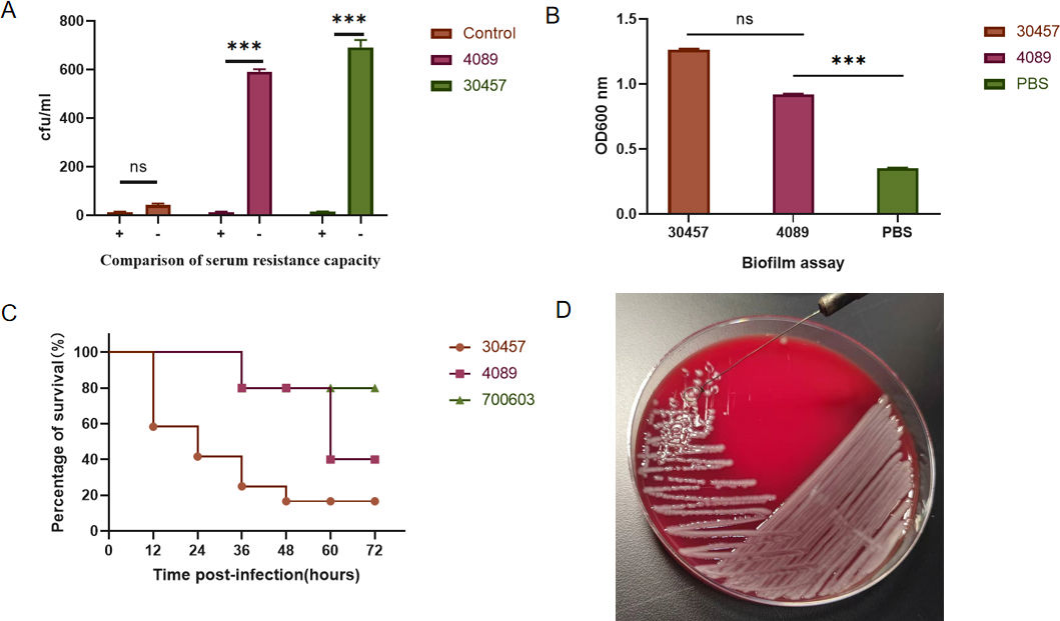
Analysis of virulence characteristics. (**A**) The serum resistance capability of KP4089. KP4089 has a weaker level of serum killing compared to hvKP30457 with ATCC700603. (**B**) Biofilm-forming ability of the strains is analyzed by the crystal violet method. (**C**) The Mann–Whitney test was used for statistical analyses. ***P < 0.001. Virulence analysis of the strain KP4089 in a Galleria mellonella infection model. (**D**) The string test of KP4089 shows that the mucus filaments＜5 mm.

The KP4089 survival rate after exposure to healthy human serum and inactivated serum was 1.37%. Control ATCC700603 and KP30457 survival rates were 28.8% and 1.88%, respectively. Statistical analysis showed that the two groups were statistically significant (*P* < 0.05) (Fig. 1A). Crystal violet staining exhibited that strain KP4089 had weaker biofilm formation than the reference group (Fig. 1B). Compared to the survival rate of KP30457, the KP4089 biofilm-forming capacity, serum killing level, and Galleria mellonella model were hypervirulent and close to that of ATCC700603 (Fig. 1C), but the string test was negative (Fig. 1D). This phenomenon indicates that the strain is a hypovirulent *Klebsiella pneumoniae*.

### Plasmid properties and fitness costs

**Fig 2:**
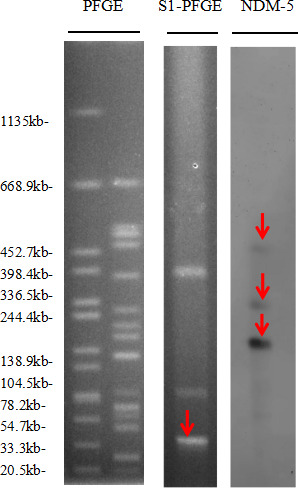
PFGE, S1-PFGE, and southern hybridization analysis of KP4089. S1 nuclease digestion of plasmid bands is shown as linearized fragments on the gel. Southern blot hybridization of the marker gene (*NDM-5*) of the resistance plasmid, marked with a red arrow. Lane H9812, reference standard strain Salmonella serotype Braenderup H9812 restricted with Xbal. Note: the red box marks KP4089.

**Fig 3:**
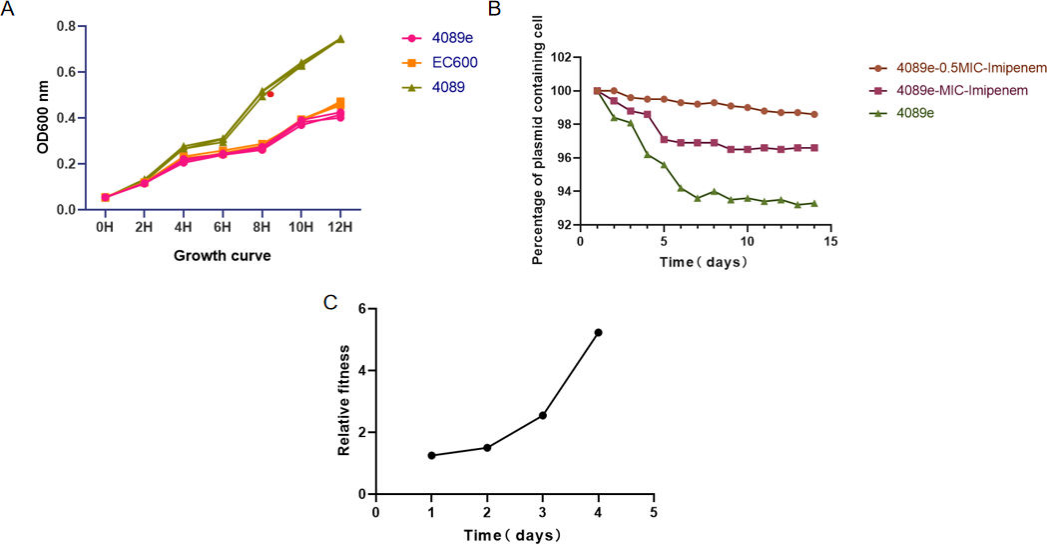
The fitness cost of the strain 4089e. (**A**) Growth curve measurements of KP4089, 4089e, and Ec600. The growth of strain 4089e was not significantly different from that of strain EC600, while the growth capacity was significantly reduced compared to strain KP4089. (**B**) Plasmid stability for the strain 4089e in 0.5MIC-Imipenem and MIC-Imipenem medium. (**C**) The relative fitness of strain 4089e. The relative fitness cost of the strain was >1.

KP4089 had drug resistance genes (*NDM-5, OXA-181, CTX-M-15, fosA6,* and *CatI*). Whole-genome sequencing revealed that the strain with the *NDM-5* gene was located on the IncN plasmid (92.284 kb), which is rare in *Klebsiella pneumoniae*. Another *OXA-181* gene was located on plasmid ColE (90.882 kb), which was highly homologous to the plasmid carrying the *OXA-181* gene from *Klebsiella pneumoniae* (accession number: CP104800). This phenomenon suggested that horizontal transfer of the plasmid occurred between or within species. S1-PFGE and southern blotting analysis showed that the strains of *NDM-5* contained three copies, each 812 bp in size, located on a plasmid 4.732 kb in size (Fig. 2). Whole-genome sequencing identified 25 resistance genes and 22 virulence genes. The strain of *NDM-5* in ST147 typing contained several insertion elements, such as ISKpn26, IS52, IS5, and IS15, which contributed to the stability of the IncN plasmid. Pre- and post-junctional drug sensitivity results showed that the transformant had a resistance phenotype comparable to that of strain KP4089, and its growth was not significantly different from that of strain EC600 (Fig. 3A). The *in vitro* growth competition experiment showed that the relative fitness cost of the strain was 1.25, indicating the IncN plasmid did not impose a fitness cost on the host strain (Fig. 3C). Plasmid stability tests showed *blaNDM-5* harboring plasmids were stable in CRKP for over 10 generations (Fig. 3B).

### Comparative genomic analysis

**Fig 4:**
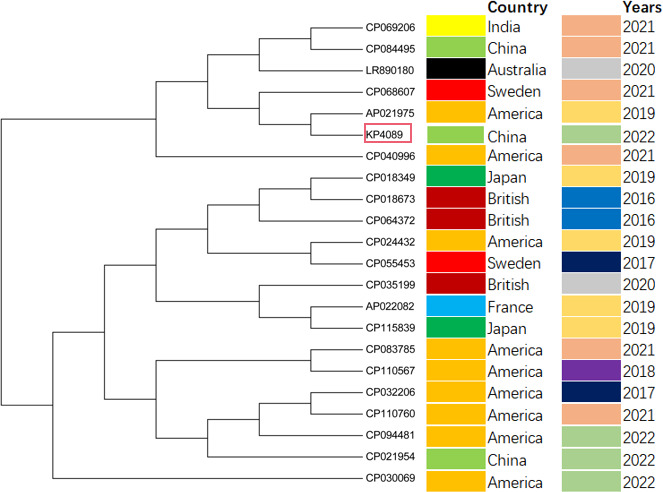
Maximum likelihood phylogeny based on the gene sequences of 22 strains of *Klebsiella pneumoniae* carrying the plasmid.

The results of the evolutionary tree analysis showed that the 22 strains of *Klebsiella pneumoniae* were mainly located in three different branches (Fig. 4). *Klebsiella pneumoniae* strains that originated in the same country were more clustered in the second and third branches, while the first branch was more dispersed. *Klebsiella pneumoniae* strains originated in the United States were found in all three branches, but they were mainly clustered in the third branch. *Klebsiella pneumoniae* KP4089 was the only *Klebsiella pneumoniae* isolate among the 22 strains known to be isolated from patients with liver abscesses (Fig. 4). KP4089 was located in the first branch and formed a cluster with five other *Klebsiella pneumoniae* strains, thus inferring six strains that were more closely related. CP069206, LR890180, CP068607, AP021975, and CP040996 were the five strains that originated in India, Australia, Sweden, and America. KP4089 was located in the neighboring evolutionary branch, with a difference in isolation time (2019 and 2022), evolutionary branches, and a similarity identity of 99.8%, respectively.

### Functional analysis of genes’ function based on whole-genome sequencing

**Fig 5:**
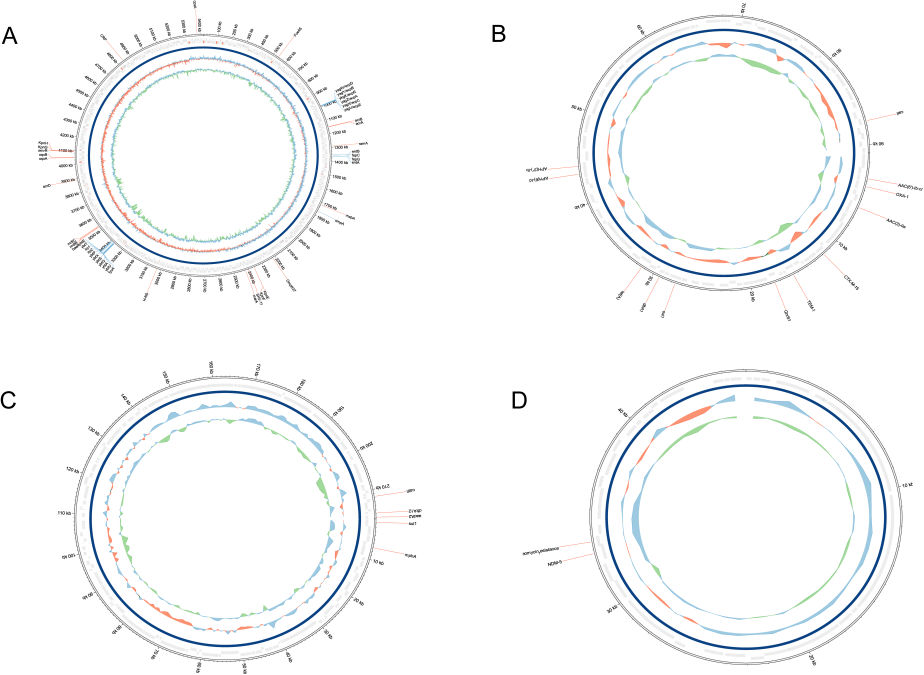
Schematic of the circular genome of KP4089 strain. Each circle contains multiple virulence and resistance genes. The outermost circle is the position coordinate of the genome sequence. (**A**) The chromosome map of strain KP4089. (**B**) Plasmid map P4089-1 of strain KP4089. (**C**) Plasmid map P4089-2 of strain KP4089. (**D**) Plasmid map P4089-3 of strain KP4089.

There was one chromosome and four plasmids in the strain. The genes *sul1*, *aadA2*, *dfrA12*, *catA2*, and *mphA* were located on plasmid P4089-1 (Fig. 5C), while the *aac(6)-Ib*, *aac(3)-IIe*, *aph(6)-Id*, *aph(3)-Ib*, *OXA-1*, *CTX-M-15*, *TEM-1*, *qnrS1*, *dfrA1*, and *tetA* genes were located on plasmid P4089-2 (Fig. 5B). The strain of *NDM-5* gene was located on plasmid P4089-3 (Fig. 5D). In addition, the *oqxA6*, *oqxB19*, *fosA*, and *SHV-11* genes were located on the chromosome (Fig. 5A), while the strain was resistant to ciprofloxacin, imipenem, piperacillin/tazobactam, tobramycin, ceftazidime/avibactam, and doxycycline.

The strain type KP4089 had ST147 multilocus sequence typing (MLST) and 64 capsular serotyping (WZI) typing. PlasmidFinder identified plasmid P4089-2 as a standard ColE-type but plasmid P4089-3 as a relatively rare IncN type. VirulenceFinder demonstrated that the strain possessed a number of virulence genes, including *ykgK/ecpR*, *yagZ/ecpA*, and *fyuA* (Fig. 5D), which were expressed in bacterial hairs, siderophores, enterobacterial transporters, and transcriptional regulators. Genomic analysis revealed that the ISKpn-21 fragment was positioned upstream of *SHV-11* and *fosA6*, showing the structure contained the genes for a breakdown enzyme (*mpkA*), an efflux pump (*oqxA6*), and an ABC transporter protein (*fepG*).

## DISCUSSION

KP is a prevalent pathogen that can infect the lungs, urinary tract, and genitalia (15). Carbapenem antibiotics are usually effective for treating KP infections. The prevalence of CRKP induced by *NDM* has been emerging, particularly for the *NDM-5* gene, complicating clinical anti-infection treatment (16). Bacterial drug-resistant plasmids have been proven to mediate strain transmission and determine drug resistance both at home and abroad (17). The IncX3 plasmid (10, 11) has been found to diversify the dissemination of the *blaNDM-5* gene to non-clonal *E. coli* hosts (ST12, ST167, ST354, ST361, ST410, ST617, ST746, ST6335, and ST6395) in China. However, it is inadequately understood that a strain with the *NDM-5* gene is present in IncN plasmids. Consequently, we analyzed the strain with the *NDM-5* gene on IncN plasmids, shedding light on its characteristics and fitness cost.

The strain (KP4089) of the *NDM-5* gene described in our study showed antimicrobial resistance to carbapenems, β-lactam, ceftazidime-avibactam, macrolides, and aminoglutethimide but remained susceptible to aminoglycosides, mucilage, and tigecycline. Furthermore, the whole-genome sequencing revealed that the strain was located on a 92.284-kb IncN plasmid and contained the drug resistance genes (*NDM-5*, *OXA-181*, *CTX-M-15*, *fosA6*, and *CatI*) along with a class I integron. These results have similarities to the previous studies (18) demonstrating the presence of class I integrons in *Klebsiella pneumoniae*. However, additional studies are needed to explore the potential mechanisms by which this bacterium is linked to multi-drug resistance.

The expression of various virulence genes in strain type KP4089 encompasses siderophore, bacteriophage hairs, and lipopolysaccharide (LPS). Previous research (19 – 21) has identified the genes ybtS, ybtX, and ybtQ as key determinants of external invasiveness. The bacteriophage function of this strain is facilitated by the *yagV/ecpE* gene, which also plays a role in promoting effective plasmid conjugation (22). The strain type KP4089 possesses certain virulence features that are associated with adhesion, such as the presence of the virulence genes *yagW* and *ecpD*. Virulence gene expression can be categorized into three classes: the iron uptake class (*entA* and *entB*), the invasive class (*ompA*), and the regulatory class (*fepC*, *fepG*). Formerly, infections caused by hvKP were commonly associated with the development of abscesses, specifically liver abscesses (23). We found that liver abscess patients’ *Klebsiella pneumoniae* samples exhibited hypovirulence and numerous genes for carbapenemase resistance. This was due to the fact that they failed string tests, reduced biofilm formation, killed much serum, and possessed a plasmid with minimal virulence. As a result, the resistance genes could be shared between strains. In view of these findings, it is of utmost importance that clinicians understand that this potentially fatal condition may manifest itself even in the absence of an active hypervirulent *Klebsiella pneumoniae* infection.

The P4089-NDM5 plasmid is a mobile genetic element capable of disseminating genes associated with virulence and drug resistance. According to the results of our conjugation assay, the strains KP4089 and EC600 yielded a transconjugant with a drug resistance phenotype similar to strain KP4089. S1-PFGE confirmed that the transconjugant contained the NDM-5 plasmid, suggesting that the transconjugant was effective. In contrast, the transconjugation of the resistance genes *OXA-181* and *tetA* failed, demonstrating that the environment influences resistance plasmid transmission. This hints that NDM plasmids have a higher propensity for dissemination compared to other resistance plasmids. Notably, the growth pattern of the strain EC600 was comparable to that of the strain with inserted IncN plasmids, providing additional evidence supporting the notion that the presence of these plasmids did not impose any constraints on the adaptability of the strain.

Lastly, our *in vitro* growth competition experiments revealed that the acquisition of IncN plasmids had no effect on the host strain’s fitness, confirming the strain’s relative fitness cost of 1.25. Nevertheless, a treatment strategy is necessary due to the rapidity with which this could spread. In addition, we discovered that *blaNDM-5* plasmids persisted for over 10 generations. This demonstrates that obtaining IncN plasmids enhances the ability of coliform bacteria to adapt to an environment with continuous transmission. The findings from our S1-PFGE and southern blot studies revealed the presence of three copies of P4089-NDM5. There are two probable reasons for this occurrence, namely the bacterium’s endogenous regulatory mechanisms and the external selective pressures exerted by the environment. This indirectly suggests that the abundance of plasmids in bacteria may serve as a potential source for the dissemination of highly resistant genes to both other bacteria and humans, thus posing a threat to the efficacy of routinely employed antibiotics for infection treatment.

### Conclusions

Whole-genome sequencing revealed that the *NDM-5* gene strain was on the IncN plasmid (92.284 kb), which is uncommon in *Klebsiella pneumoniae*. The drug resistance genes (*NDM-5*, *OXA-181*, *CTX-M-15*, *fosA6*, and *CatI*) were found in the strain KP4089, together with a class I integron. The relative fitness cost of the strain was greater than 1 in the *in vitro* growth competition experiment, indicating that the IncN plasmid did not impose a fitness cost on the host strain. To prevent the spread of hypovirulent multidrug-resistant KPs, continuing clinical surveillance of such strains must be increased, and future research should focus on determining the potential strain and mechanistic studies.

## Data Availability

The genome sequence of KP4089 was submitted to GenBank for the first time under the accession number SRR23012586.

## SUPPLEMENTAL MATERIAL

Supplemental material spectrum.03443-23-s0001.txt Whole genome sequencing.

Table S1 spectrum.03443-23-s0002.docx The sequence of Intl.

ASM does not own the copyrights to Supplemental Material that may be linked to, or accessed through, an article. The authors have granted ASM a non-exclusive, world-wide license to publish the Supplemental Material files. Please contact the corresponding author directly for reuse.
