# Supplementary material for: Analysis of the hypovirulent Klebsiella pneumoniae with the NDM-5 gene on IncN plasmids
Source: Microbiol Spectr. 2023 Nov 29;12(1):e03443-23. doi: 10.1128/spectrum.03443-23 (PMC10783101; doi:10.1128/spectrum.03443-23)
Supplement: Table S1 — The sequence of Intl. [file spectrum.03443-23-s0002.docx]

Table S1.Integron primer sequence table

| Primer | [Primer Sequence](javascript:;)（5’-3’） | [Length](javascript:;)（bp） |
| --- | --- | --- |
| IntI1F | ACGAGCGCAAGGTTTCGGT | 565 |
| Intl1R | GAAAGGTCTGGTCATACATG |  |
| IntI2F | GTGCAACGCATTTTGCAGG | 403 |
| IntI2R | CAACGGAGTCATGCAGATG |  |
| Intl3F | CATTTGTGTTGTGGACGGC | 717 |
| IntI3R | GACAGATACGTGTTTGGCAA |  |
